# Microbes on the Mobile Phones of Healthcare Workers in Palestine: Identification, Characterization, and Comparison

**DOI:** 10.1155/2021/8845879

**Published:** 2021-02-26

**Authors:** Mohammad Qadi, Rasha Khayyat, Mohammed A. AlHajhamad, Yazan I. Naji, Beesan Maraqa, Kais Abuzaitoun, Ahmed Mousa, Maysa Daqqa

**Affiliations:** ^1^Department of Biomedical Sciences, Faculty of Medicine and Health Sciences, An-Najah National University, Nablus, P.O. Box 7, State of Palestine; ^2^Department of Medicine, Faculty of Medicine and Health Sciences, An-Najah National University, Nablus, P.O. Box 7, State of Palestine; ^3^Primary Health Directorate, Palestinian Ministry of Health, Nablus, State of Palestine

## Abstract

**Background:**

Healthcare workers (HCWs) may be using their mobile phones (MPs) to carry microbes that cause hospital-acquired and community infections in general. With antibiotic resistance problem emergence, these infections can be challenging to eradicate. Hence, this study aimed to determine the microbial contamination of HCW MPs and identify and classify bacterial isolates in Palestine.

**Methods:**

This was a 7-month comparative cross-sectional analysis of 200 HCW MPs from 2 hospitals and 100 MPs from university students (non-HCWs). Data collection was done using a self-administrated questionnaire, and a swab sample from both HCW and non-HCW MPs was obtained and transferred to An-Najah National University (NNU) microbiology lab for bacterial identification and antibiotic susceptibility. Data were analyzed using Social Sciences Statistical Package (SPSS) version 22.0.

**Result:**

Among HCWs, the microbial contamination was 87.5%. Coagulase-negative *staphylococci* (CoNS; 67.3%), methicillin-sensitive *Staphylococcus aureus* (MSSA; 17.5%), Gram-positive bacilli (4.1%), methicillin-resistant *Staphylococcus aureus* (MRSA; 1.6%), and Gram-negative species (1.6%) were the most predominant bacterial isolates. More than half of *staphylococci* isolates were resistant to penicillin and erythromycin. Male gender, using a mobile phone in the bathroom, and entry to the operating theatre were associated with mobile phone contamination and increased resistance against specific antibiotics. Among non-HCWs, the contamination was 86%. The most predominant bacterial isolates were CoNS, MSSA, and Gram-positive bacilli, with a contamination of 66.8%, 28.5%, and 2.6%, respectively. No MRSA or Gram-negative species were detected in this group. Antibiotic resistance percentage of *staphylococci* was nearly half of that yielded in the HCW group against each antibiotic.

**Conclusion:**

Significant numbers of bacteria have been isolated from HCW MPs. Working in a hospital environment frequently raises the probability of presence of antibiotic-resistant bacteria on a MP. Therefore, infection control teams should discuss methods to prevent the transmission of drug-resistant pathogens from HCW MPs.

## 1. Introduction

A nosocomial infection (NI) is a severe global problem and contributes significantly to patient morbidity and mortality [1]. Mobile phones (MPs) are essential in everyday life and are carried everywhere and at all times [2]. Moreover, they are always in close contact with the hands and body skin [3]. MPs are also becoming an essential tool in medical practice. First, they are primarily used in communication among healthcare workers (HCWs), making healthcare work more efficient [4]. Secondly, they provide medical information access by asking for consultation and reaching medical references [2]. However, MPs are rarely disinfected and are often touched by HCWs before, during, and after examining their patients without handwashing [3]. Many studies reported the colonization of potentially pathogenic organisms on various objects as hands, MPs, personal digital assistants, computer keyboards, and pagers. These objects have been suggested as possible vectors for the transmission of nosocomial pathogens from HCWs to patients as well as severe acute respiratory syndrome coronavirus 2 (SARS-CoV-2) [5–8]. Contaminated MPs are potential vectors for spreading microbes in both hospitals and communities, making them an exogenous source of nosocomial infections [9–12].

The NI affects vast numbers of patients globally, significantly raising mortality rates and financial losses. Figures show that about 15% of all hospitalized patients suffer from these infections. In high-income countries, the incidence is high enough, between 3.5% and 12%, while in middle and low-income countries (LMICs), such as Palestine, it varies from 5.7% to 19.1% [13]. NIs caused by multidrug resistant bacteria are a growing problem in many healthcare institutions [13]. Instruments, hands, MPs, and other inanimate hospital objects used by the HCW may serve as vehicles and reservoirs for the NIs. However, the incidence of such infections can be reduced by maintaining proper hygiene between the HCW and the hospital environment [1, 14, 15].

MPs have become multipurpose nonmedical devices used in the healthcare facility and the community. They have increasingly become an essential means of communication in the community and healthcare facilities to collect epidemiological data and monitor chronic diseases [15, 16]. MPs are used without restriction in healthcare facilities regardless of their microbial load. Attempting to avoid NIs, it is worth studying and identifying pathogens on MPs to improve the quality of healthcare. MPs' potential to become a nosocomial infection source has been studied before [6, 17, 18]. However, to the best of our knowledge, no study or published data have been done in Palestine. This research aims to analyze and compare the degree of contamination of HCW members with non-HCW members. In addition, it aims to examining the prevalence of antibiotic-resistant bacteria on MPs' and correlate it to handling habits.

## 2. Materials and Methods

### 2.1. Study Design and Setting

This is a comparative cross-sectional study of the frequency of bacterial contamination of HCW and non-HCW participants between September 2018 and March 2019. This study was performed in Nablus, situated in the northern part of the West Bank, Palestine.

### 2.2. Study Population

In this study, the participants' MPs were sampled without any prior intimation and divided into two groups: the HCW group and the non-HCW group. We recruited HCW participants from two separate hospitals in Nablus, West Bank, Palestine: Rafidia Surgical Hospital as a governmental hospital and An-Najah National University Hospital (NNUH) as a nongovernmental hospital. We considered the governmental hospital as hospital A and the nongovernmental hospital as hospital B. On the other hand, the non-HCW group comprises engineering and IT students at An-Najah National University (ANNU) since their field depends on using MPs more frequently and they have no interaction with the healthcare system during their study.

#### 2.2.1. Inclusion Criteria

HCWs were physicians, residents in training, nurses, and any other person who has a hospital position as lab technicians and radiology staff. Consenting participants were asked to enroll their phones, choosing the most frequently used device, in case of multiple eligible devices.

#### 2.2.2. Exclusion Criteria

We excluded personnel who do not possess or use MPs at the time of analysis and those who cleaned their phones once they heard about our study, and those whose MPs were contaminated by our hands during sample collection were also excluded.

### 2.3. Sample Size

By considering the following statistical assumptions, the sample size for this analysis uses a double population proportion formula: 95% confidence level, the prevalence of contamination is 50% and 5% error margin, and a power of 80%. Rosoft sample size calculator was used. The total population of healthcare staff in both hospitals was 400, and half of the HCW's contamination was anticipated in non-HCW's. As a result, samples of 200 from MPs of HCW and 100 from MPs of non-HCW were enrolled.

#### 2.3.1. Questionnaire

Consent participants were asked to complete a questionnaire before taking a swab sample from their MPs. The questionnaire addressed participants' usage habits by several domains: their daily use and a special section asking about using their MPs in hospital and medical practice. Only the HCW has filled this particular section. The questionnaire was written in the local language, Arabic. It included questions about gender, age, and average time spent daily on MPs. Other questions assessed the frequency of handwashing per day, frequency of MP cleaning per month, last time of MP cleaning, and MP cleaning method. Besides, questions set smoking status, using MP in the bathroom, and broken quality of the screen or the screen protector.

A special section asked HCWs about their position at the hospital, availability of antiseptic solutions in the wards, using mobile on the entrance to operating rooms, using the MPs in the operating theatre, and using MPs next to the patient.

To ensure its validity and reliability, the questionnaire was pretested before the finalization. The questionnaire was checked by experts from An-Najah National University Faculty who suggested adding several questions.

A pilot study was performed on 20 samples for both training purposes on the sampling process and to check the questionnaire's simplicity and readability and the time it takes to be filled.

#### 2.3.2. Sample Collection

A sterile cotton swab with a transport medium was used to take a swab from the touchscreen, mouthpiece, earpiece, and buttons since these areas are the most frequent spots in contact with the fingers, as done in other studies [3]. After swabbing, samples were transferred immediately and adequately within 2 hours to the NNUH microbiology lab for culturing on suitable media (blood agar and MacConkey). Then, the inoculated media were incubated at 37°C for 48 hours.

#### 2.3.3. Identification of Microorganisms

After incubation time was over, colonies that grew on plated media were subcultured to obtain a pure culture from each isolated type of colony. Then, isolated microorganisms were identified using different microbiological identification approaches, including morphological and biochemical features.

Briefly, all isolates were subjected to Gram stain, and based on the result, identification was performed as follows:

- A spore staining procedure was applied for such isolates as Gram-positive bacilli. This was for allowing differentiation into spore-forming and non-spore-forming Gram-positive bacilli.
- For Gram-positive cocci, isolates were subjected to catalase test, and those with positive reaction were cultured on mannitol salt agar to classify them into *S. aureus* and CoNS. At the same time, catalase-negative isolates were considered as other Gram-positive cocci.
- A glucose fermentation test was applied for Gram-negative bacilli to group them into glucose fermenters and glucose nonfermenters.

Finally, based on the mentioned adapted identification scheme, isolated microbes were grouped for data analysis.

#### 2.3.4. Antibiotic Sensitivity Test

This test was performed by the Kirby–Bauer disc diffusion method on Mueller-Hinton agar according to Clinical Laboratory Standards Institute 2018 antibiotic disc susceptibility testing guidelines. Antibiotic sensitivity profile was done only for *Staphylococcus* species. Antibiotics used for this group of bacteria were as follows: penicillin (P), clindamycin (DA), cefoxitin (FOX), erythromycin (E), and trimethoprim-sulfamethoxazole (SXT). *S. aureus* isolates resistant to FOX were considered as methicillin-resistant *S. aureus* (MRSA) and were, afterward, tested with 30-microgram vancomycin disc on Mueller-Hinton agar to test for the existence of vancomycin-resistant *S. aureus*.

### 2.4. Data Analysis

The Statistical Package of Social Sciences (SPSS) v. 22.0 software was used to analyze the obtained data. Using tables and figures when needed, a descriptive analysis of sample results was presented. Using the chi-square test, the association between research variables was evaluated. The agreed significance level has been set below 0.05 (*p* < 0.05).

### 2.5. Ethical Consideration

Approval was obtained from the IRB (Institutional Review Board) at NNU before initiating this research. The privacy and confidentiality of the participants were ensured. After participants read the research overview, informed consent was obtained orally, and the participants were informed that their involvement in the study was voluntary.

## 3. Results

In this study, 200 samples of HCWs and 100 samples of non-HCWs were obtained. Of those who completed the questionnaire, more than half of the participants were males, as shown in Table 1.

MP-related habits and comparisons of the questionnaire data of HCWs with non-HCWs are presented in Table 1. Daily time spent on the phone showed considerable variation between the two groups; spending more than two hours on MPs was more common among HCWs. On the other hand, the handwashing level of HCWs was substantially higher than that of non-HCWs. The use of alcohol in parliamentary cleaning was considerably more common among HCWs.

Samples of HCWs were collected from specialists (10.6%), nursing staff (44%), medical residents (28.2%), and other healthcare professionals such as technicians working in various departments: laboratory, radiology department, optometry, operating theatre, and general wards (13.6%). Relevant scenarios in which the HCW could use their MPs and contribute NIs were described in the questionnaire, as shown in Table 2. HCWs demonstrated that most of them insisted on the availability of hand sanitizer at all times in the facility they serve. When entering the operating room, doctors (specialists and residents) were more likely to use their MPs, while residents were much more likely to use their mobile phones alongside patients.

### 3.1. Prevalence and Type of Bacterial Isolates

A total of 300 mobile phone samples were examined for the presence of bacterial contamination. Bacterial contamination was found in swabs taken from 175 HCW MPs (87.5%) and 86 non-HCW MPs (86%). No significant difference was noticed in the study's microbial contamination between the HCW and control group non-HCW. The total number of bacterial isolates was 628 from both groups.

**Figure 1:**
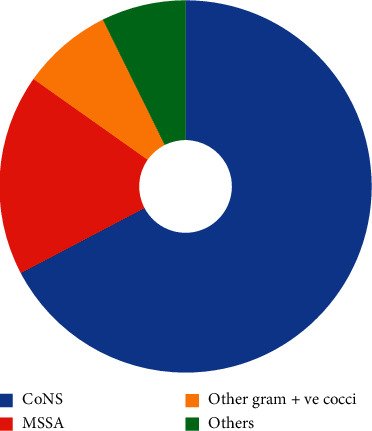
Distribution of bacterial isolates from HCW MPs.

From the 200 HCW mobile phones, 435 bacterial isolates were obtained and characterized.  Figure 1 illustrates the different types of bacteria isolated and their distribution. Four hundred twenty-eight bacterial isolates were found to be Gram-positive. Among which, 293 bacteria were CoNS (67.3%), 76 were methicillin-sensitive *Staphylococcus aureus* (MSSA) (17.5%), 13 were non-spore-forming Gram-positive bacilli (3%), 5 were spore-forming Gram-positive bacilli (1.2%), and 34 were other Gram-positive cocci (7.9%). Seven bacterial isolates were found to be Gram-negative bacteria (1.6%), among which 3 were glucose fermenters (0.7%) and 4 were non-glucose fermenters (0.9%).

Table 3 illustrates the significant differences between hospitals A and B concerning isolated bacteria. No significant differences were noted between the two hospitals. In hospital A, MRSA, Gram-negative species, and Gram-positive bacilli percentages were higher.

**Figure 2:**
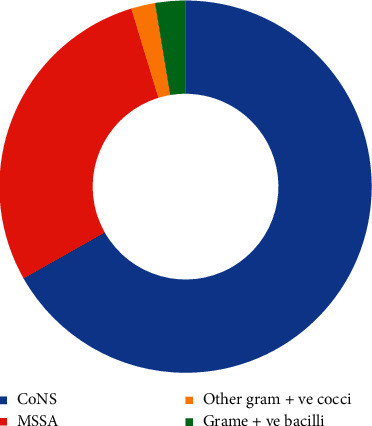
Distribution of bacterial isolates from non-HCW mobile phones.

From the 100 non-HCW MPs, 193 bacteria were isolated. Interestingly, those isolates were found to be only Gram-positive bacteria. Among which, 129 were CoNS (66.8%), 55 were MSSA (28.5%), 5 were non-spore-forming Gram-positive bacilli (2.6%), and 4 were other Gram-positive cocci (2.1%). There were no MRSA, no spore-forming Gram-positive bacilli, or Gram-negative bacteria isolated. The distribution of bacteria from the non-HCW is shown in Figure 2.

### 3.2. Antibiotic Susceptibility Pattern

Antibiotic susceptibility pattern of *Staphylococci* isolated from HCW and non-HCW MPs is shown in Table 4. Regarding the susceptibility of *S. aureus* in HCW MPs, cefoxitin was the most effective antibiotic (susceptibility = 82.1%). Other antibiotics came next: clindamycin (*S* = 67.2%), trimethoprim-sulfamethoxazole (*S* = 64.2%), erythromycin (*S* = 23.9%), and penicillin, which was the least influential (*S* = 14.9%). For CoNS, cefoxitin was also the most effective antibiotic (*S* = 91.3%). It was followed by trimethoprim-sulfamethoxazole (*S* = 72.5%), clindamycin (*S* = 64.9%), erythromycin (*S* = 23.5%), and penicillin, which was the least influential (*S* = 19.5%).

Furthermore, for *S. aureus* in non-HCWs, cefoxitin was the most effective antibiotic (*S* = 96.4%). For trimethoprim-sulfamethoxazole, it was *S* = 85.7%, clindamycin showed *S* = 78.6%, and penicillin and erythromycin showed the same efficacy (*S* = 37.7%). For CoNS, cefoxitin was also the most effective antibiotic (*S* = 96.5%), followed by trimethoprim-sulfamethoxazole (*S* = 75.9%), clindamycin (*S* = 62.1%), penicillin (*S* = 31.7%), and erythromycin, which was the least influential (*S* = 26.3%).

It is quite clear that there are reductions in all antibiotics' susceptibility, shown by *Staphylococcus* isolated from the HCW MPs compared to that separated from the non-HCW. Data are more precise in *S. aureus* than CoNS.

The differences between hospital A and B concerning resistance to antibiotics are shown in Table 5. Bacterial isolate resistance against clindamycin, cefoxitin, and trimethoprim-sulfamethoxazole was more in hospital A. However, resistance against penicillin and erythromycin was more in hospital B.

MP contamination differs in distribution according to a hospital position as shown in Table 6; the highest percentage of contamination was among the residents (100%), and the lowest was among the technicians (70%).

## 4. Discussion

Many published articles highlighted the role of MPs as a pathway of microbial transmission. The latest systematic analysis of MP microbial contamination reveals a high overall prevalence of contamination from healthcare and community environments, hitting 68 percent [7].

More than eighty percent of HCW MPs showed microbial growth in the current study. Several other studies documented different contamination levels. Other studies performed in LMICs have demonstrated vast contamination levels [19, 20]. Higher findings of bacterial contamination were also reported in Taiwan (94.3%) [2], Ethiopia (94.2%) [15], United Arab Emirates (98%) [21], and Turkey (97.8%) [10]. Also, our findings indicate that bacteria contaminate 86% of non-HCW MPs. This contamination level is close to research by Neha et al. [22].

Factors associated with contamination of MPs were MP owner's gender. Male HCW MPs were more contaminated, where male to female ratio was 1.5 : 1. This result is similar to a study conducted in Ethiopia [15] and Iran [23]. HCW MP-using habits showed essential associations with the contamination of MPs. Using MPs in the bathroom was associated with contamination by Gram-positive bacilli (*p* value = 0.026).

Other data findings from the questionnaire on the MP usage behaviors, such as frequency of handwashing per day, frequency of cleaning of MPs per month, last time cleaning of MPs, cleaning method of MPs, smoking status, time spent using mobile phones per day, and hand washing after use of MPs, were checked for any correlation with laboratory results. None of these behaviors were correlated with any association.

The investigation of bacteria isolated from HCW MPs revealed that CoNS were the majority of the bacteria. The CoNS colonization average was up to 67.3% in these samples. This outcome is consistent with other studies, such as Kokate et al. [24], Pal et al. [3], and Bodena et al. [15]. CoNS have relatively low virulence and tend to be the skin's natural flora. However, these bacteria have become increasingly recognized as the most common cause of nosocomial bacteremia associated with indwelling devices [14]. In this study, CoNS isolate percentage was lower than reported in Taiwan (90.2%) [2] and in India (72%) [24]. However, these isolates' percentage was higher than Egypt's results, which showed 50% [14].

The second most common bacterial isolate from HCW MPs was *S. aureus*. This percentage was 17.5% MSSA and 1.6% MRSA. Both MSSA and MRSA are potential clinical pathogens. *S. aureus* can cause various illnesses, from minor skin infections to much more severe diseases. MRSA is of particular importance in the medical community, as it has evolved resistance to *β*-lactam antibiotics [25]. MRSA was detected on HCW stethoscopes and MPs' [26]. Tambe and Pai study in India [27]and in a community study in Italy [28] both reported that the isolation of *S. aureus* was the highest in all the categories of HCWs, 54% and 64.1%, respectively. The genus of *Staphylococcus* was the most commonly observed isolates. It is primarily due to their ability to tolerate dryness and thus thrive and multiply rapidly in warm environments such as MPs [28].

For the other Gram-positive cocci, we found 7.9%. Our findings were similar to those of Egypt [14], whereas Tambe and Pai found a higher percentage of other Gram-positive cocci in Turkey [27]. As for Gram-positive bacilli (4.2% of total isolates), 1.2% were spore forming, and 3% were non-spore forming. In comparison, the Gram-negative bacilli were 1.6% of the total isolates, and this is considerably lower than other Ethiopia studies, where *Klebsiella* has been identified as the third most prevalent pathogenic bacteria [15].

As for the non-HCW group, CoNS were also the most prevalent (66.8% of total isolates), similar to a study by Bhoonderowa et al. [28]. CoNS were also the most prevalent (69%) bacteria from MPs of non-HCWs in Misgana et al., which reported a prevalence of 56% [29]. As for the second most common non-HCW isolated bacteria, we found MSSA to be 28.5%. Non-HCW outcomes were more outstanding than the HCW MPs, with no MRSA observed on their MPs. For the third most common isolate, the percentage of Gram-positive non-spore-forming bacilli was 2.6%. Additionally, 2.1% of total isolates comprised other Gram-positive cocci. No Gram-negative bacilli or Gram-positive spore-forming bacilli were isolated.

Antimicrobial resistance is the most severe health threat in treating patients (WHO). Our results showed that CoNS isolated from HCW MPs were more susceptible to cefoxitin (*S* = 91.3%). For trimethoprim-sulfamethoxazole, the percentage was *S* = 72.5%, clindamycin showed *S* = 64.9%, erythromycin showed *S* = 23.5%, and penicillin showed *S* = 19.5%. Regarding cefoxitin, our results showed better susceptibility than another study conducted in Iran [30] that showed *S* = 52.3%. In contrast, CoNS in the same study had better susceptibility to erythromycin (*S* = 43.1%) and clindamycin (*S* = 71.4%) compared to our results. Among *S. aureus* isolated from the same above study group, cefoxitin was also the most effective antibiotic (*S* = 82.1%), followed by clindamycin (*S* = 67.2%), trimethoprim-sulfamethoxazole (*S* = 64.2%), erythromycin (*S* = 23.9%), and penicillin, which was the least effective (*S* = 14.9%). In our study, the prevalence of MRSA in the HCW group was 17.9%. Our results showed better *S. aureus* susceptibility to cefoxitin than the Iranian study that showed *S* = 52.3%. In contrast, the same study had better susceptibility to erythromycin (*S* = 43.1%) and clindamycin (*S* = 73.4%) than our results. This difference in antibiotic susceptibility compared to other studies might be due to different hospital environments, bacterial strains, empirical treatment practices, easy access to antibiotics without a prescription, and prolonged use of common antibiotics [30, 31].

On the other hand, isolates from non-HCW MPs showed less resistance to the last antibiotics than the HCW group. CoNS isolated from non-HCW MPs were all susceptible to cefoxitin (*S* = 100.0%). Results for other antibiotics were as follows: trimethoprim-sulfamethoxazole (*S* = 75.9%), clindamycin (*S* = 62.1%), penicillin (*S* = 31.7%), and erythromycin, which was the least influential (*S* = 26.3%). Among *S. aureus* isolated from the non-HCW group, cefoxitin was also the most effective antibiotic (*S* = 96.4%). For others, trimethoprim-sulfamethoxazole showed *S* = 85.7%, clindamycin showed *S* = 78.6%, and penicillin and erythromycin showed the same efficacy (*S* = 37.7%). No MRSA isolates were detected in the non-HCW group compared to 17.9% of *S. aureus* in the HCW group.

By connecting answers to the questionnaire and antibiotic resistance profile of isolates, we found that MP-using habits of HCWs were associated with increased bacterial resistance against specific antibiotics. Cefoxitin resistance was related to the existence of screen breaks on the MPs (*p* value = 0.042), entry to operating theatre (*p* value = 0.035), and increased average use of MPs per day (*p* value = 0.011). Erythromycin resistance was also associated with entry to operating theatre (*p* value = 0.007). Trimethoprim-sulfamethoxazole resistance was associated with using MPs in the bathroom (*p* value = 0.003) and being a nurse or resident in a hospital position (*p* value = 0.036).

By comparison, more significant numbers of different isolates were isolated from MPs of the HCW than that of the non-HCW. Working in a hospital environment also raised the possibility of bacterial antibiotic resistance. This finding is probably due to the hospital environment and contact with patients with different infections. This increase of antibiotic resistance found in isolates from HCW MPs indicates that MPs are very likely to increase the burden of NIs. Never the less, it was found that HCWs cleaned their MPs with alcohol more frequently than non-HCWs as shown in Table 1. Not all is positive, as among HCWs, it was found that residents used their MPs in healthcare settings the most, whether it is on the entry or inside the operating theatre or beside patients. That is why it is essential to publish mandatory guidelines on the use and cleaning of MPs in healthcare settings and to take measures to reinforce them. It is interesting to mention that to present, no guidelines on the cleaning of MPs and restrictions of their use in our healthcare setting in Palestine exist.

Cross-sectional design limitations may have an impact on this research. Report bias could be introduced, by having MP handling data reported by self-reported questionnaires. Samples were obtained in all shifts on all days of the week to minimize selection bias, and it was a random process as we chose that we meet in different hospital shifts. Fortunately, because of being busy or in a hurry, only two nonresponders have been detected by the data collection process.

## 5. Conclusion

Our study shows that more significant numbers of organisms were isolated from MPs of HCWs than those of non-HCWs. CoNS were the most frequently identified bacteria contaminating MPs. It is known to be positively associated with nosocomial bacteremia. Our findings indicate that cefoxitin is the most effective antibiotic for most bacteria isolated from contaminated MPs. Being a male or a nurse, working at an operating theatre, and being aware of MPs role in spreading microbes were associated with higher contamination percentages. Also, using MPs in bathrooms increases the risk of transmission of Gram-positive bacilli. Working in a hospital environment seems to raise the possibility of bacterial antibiotic resistance.

From what precedes and from other previous studies, it looks like HCW constitutes a significant epidemiologic hazard to the public by microbial contamination of their MPs. Regular decontamination of MPs with alcohol wipes and proper hand hygiene may decrease the risk of hospital-acquired infections caused by these devices [6, 32]. Hospitals should limit MP use in the operating theatre and encourage the use of specific fixed MPs instead of personal ones to promote the application of mobile hygiene [11]. Moreover, disposable sterile MP pouches while working beside patients would help decrease MP bacterial contamination.

Additionally, it is worth studying the contamination of other instruments and devices frequently used by HCWs, such as stethoscopes and computer keyboards, by pathogenic bacteria. This would help rule out other sources of infection and contribute to stopping the cycle of nosocomial infections and saving lives [12].

## Figures and Tables

**Table 1:** Background variables of the participants.

Variable	Participant characteristic		*p* value
	Non-healthcare worker (*n* = 98)	Healthcare workers (*n* = 195)	
	Frequency (%)	Frequency (%)	
Gender			
Male	52 (53.1%)	111 (56.9%)	0.63
Female	46 (46.9%)	84 (43.1%)	
Missing	0 (0%)	0 (0%)	
Time spent using the phone on average			
<60 minutes	12 (12.2%)	67 (34.4%)	<0.001
60–120 minutes	18 (18.4%)	41 (21.0%)	
>120 minutes	68 (69.4%)	86 (44.1%)	
Missing	0 (0%)	1 (0.5%)	
Number of times of handwashing daily			
≤Four times	32 (32.7%)	29 (14.9%)	<0.001
5–7 times	33 (33.7%)	42 (21.5%)	
>7 times	32 (32.6%)	123 (63.1%)	
Missing	1 (1%)	1 (0.5%)	
Frequency of phone cleaning			
Daily	09 (9.2%)	48 (24.6%)	0.008
Weekly	38 (38.8%)	76 (39%)	
Monthly	16 (16.3%)	21 (10.8%)	
Rarely	34 (34.7%)	48 (24.6%)	
Missing	1 (1%)	2 (1%)	
Last time phone cleaning			
Last three days	37 (37.8%)	77 (39.4%)	0.97
3–6 days ago	21 (21.4%)	39 (20.0%)	
A week or more	18 (18.4%)	33 (17.0%)	
A month or more	22 (22.4%)	45 (23.1%)	
Missing	0 (0%)	1 (0.5%)	
Method of cleaning the phone			
Alcohol	11 (11.2%)	112 (57.4%)	<0.001
Water	15 (15.3%)	6 (3.1%)	
Wet wipes	52 (53.1%)	48 (24.6%)	
Paper wipes	20 (20.4%)	25 (12.8%)	
Missing	0 (0%)	4 (2.10%)	
Smoking			
Yes	11 (11.2%)	46 (23.6%)	0.017
No	86 (87.8%)	149 (76.4%)	
Missing	1 (1%)	0 (0%)	
Use of phone in the bathroom			
Yes	45 (45.9%)	78 (40.0%)	0.36
No	50 (51.1%)	112 (57.5%)	
Missing	3 (3%)	5 (2.5%)	
Breaks in the screen or screen protector			
Yes	38 (38.8%)	79 (40.5%)	0.58
No	57 (58.1%)	110 (56.4%)	
Missing	3 (3.1%)	6 (3.1%)	

**Table 2:** The use of MPs by HCWs in unique characteristic scenarios.

Scenario	HCW				

	Specialist	Resident	Nurse	Other HCWs	*p* value^*∗*^
	(*N* = 21)	(*N* = 56)	(*N* = 86)	(*N* = 27)	
	*n* (%)	*n* (%)	*n* (%)	*n* (%)	
The constant presence of hand sanitizer in the medical facility^*∗∗*^					
Yes	19 (90.3)	49 (87.5)	84 (96.6%)	25 (92.6%)	0.126
No	2 (9.5)	7 (12.5)	2 (2.40%)	2 (7.4%)	
Using mobile on entry to operating theatre^*∗∗*^					
Yes	12 (57.1)	38 (67.9)	33 (38.4)	5 (18.5)	<0.001
No	9 (42.9)	18 (32.1)	53 (61.6)	22 (81.5)	
Usage of mobile phone in the operating theatre^*∗∗*^					
Yes	7 (33.3)	23 (41.1)	10 (11.8)	3 (11.1)	<0.001
No	12 (57.1)	31 (55.4)	72 (83.5)	20 (74.1)	
Usage of the mobile phone next to patients^*∗∗*^					
Yes	7 (33.3)	34 (60.7)	34 (39.5)	10 (37.0)	0.026
No	14 (66.7)	22 (39.3)	52 (60.5)	16 (59.2)	

^*∗*^Chi-square test, ^*∗∗*^missing = 10.

**Table 3:** Comparison of bacterial isolates between hospitals A and B.

	Rafidia Hospital (hospital A)		NNUH (hospital B)	
	Percentage (%)	Number	Percentage (%)	Number
Number of samples		100		100
Total number of isolates	—	212	—	223
Contamination	87	-	88	-
Distribution of bacterial isolates				
Gram-positive cocci:	**92.9**	**197**	**95.5**	**213**
*Staphylococci* species	83.0	176	89.7	200
CoNS	64.2	136	70.4	157
*S. aureus*	18.9	40	19.3	43
MSSA	17	36	17.9	40
MRSA	1.9	4	1.3	3
Other gram-positive cocci	9.9	21	5.8	13
Gram-positive bacilli:	**5.2**	**11**	**3.1**	**7**
Non-spore forming	3.8	8	2.2	5
Spore forming	1.4	3	0.9	2
Gram-negative species:	**1.9**	**4**	**1.3**	**3**
Non-glucose fermenter	1.4	3	0.4	1
Glucose fermenter	0.5	1	0.9	2

**Table 4:** Antibiotic susceptibility pattern of *Staphylococci* isolated from HCW and non-HCW MPs.

Antibiotics	*Staphylococcus aureus*		Coagulase-negative *Staphylococcus*	
	HCWs	Non-HCWs	HCWs	Non-HCWs
	Susceptible (%)	Susceptible (%)	Susceptible (%)	Susceptible (%)
Penicillin	14.9	35.7	19.5	31.7
Clindamycin	67.2	78.6	64.9	62.1
Cefoxitin	82.1	100	91.3	96.5
Erythromycin	23.9	35.7	23.5	26.3
Trimethoprim-sulfamethoxazole	64.2	85.7	72.5	75.9

**Table 5:** Antibiotic resistance of hospital A and B bacterial isolates.

Antibiotic resistance	Rafidia Hospital (hospital A) (%)	NNUH (hospital B) (%)
Penicillin	54.2	64.5
Clindamycin	22.6	15.5
Cefoxitin	5.1	2.50
Erythromycin	52.8	59.0
Trimethoprim-sulfamethoxazole	15.5	13.0

**Table 6:** Contamination distribution according to the position in the hospital.

Position	MP contamination (%)
Specialists	90.5
Residents	100.0
Interns and med. students	87.5
Nurses	87.4
Technicians	70.0
Others	83.3

## Data Availability

The data used to support the findings of this study are included within the article.
